# Hæmorrhage in the Newly-Born

**Published:** 1907-07-20

**Authors:** 


### Diseases of Children.

### HÆMORRHAGE IN THE NEWLY-BORN.

Accidental hæmorrhage may take place from the umbilical cord or from the navel after separation of the cord. Traumatic hæmorrhage results from injury during labour, and takes place into the cranial cavity and viscera, or subcutaneously as cephalhæmatoma. But there is another variety which is in many respects more difficult to explain and very fatal. It is sometimes spoken of as the hæmorrhagic disease of the newborn, or, according to its frequent site, as hæmatemesis and melæna neonatorum. It may be defined as a spontaneous capillary oozing, which begins shortly after birth and lasts for a few days to a few weeks.

Probably it is more common than supposed, for these cases are more likely to come under the care of general practitioners than to be seen by consultants or hospital physicians, and are consequently not often reported. The following two cases are fairly typical instances of the affection. A minute baby, one month premature, was brought to hospital for melæna which had begun on the seventh day of life. The blood was rather dark, becoming lighter later on. A few clots were present. Death resulted from asthenia three days after admission. The second case was seen in consultation for melæna. The child was the third one, of healthy parents, and very well nourished. The bleeding began on the seventh day, was profuse on the eighth, and resulted in death two days later. The bleeding was profuse, of bright red watery blood, passed frequently, without clots and with little fæcal matter. There was no jaundice or fever.

Hæmorrhage of this nature may take place from the umbilicus; the mucous membrane of the nose, mouth, alimentary tract, and the genito-urinary system; from any abrasion of the skin, *sub cutem*, or even through the skin of the palms and soles; into the various viscera and cavities of the body; on the surface and into the parenchyma of different organs. Some cases of suprarenal hæmorrhage are of this type.

### Causation and Course.

The etiology is doubtful. Males seem more liable than females. Some are delicate and premature, while others appear normal infants. Hereditary syphilis has been blamed, and was present in two out of twelve cases (Abt, 1903). Endarteritis of small vessels has been found, and may be syphilitic in origin. On the other hand, in many cases there is nothing to suggest the possibility of syphilis. Perhaps such an infection increases the liability. An important predisposing factor is the sudden change which takes place in the circulatory system at birth, producing general hyperæmia of the mucous membranes throughout the body and in the internal organs. Thus, bleeding from a mucous membrane may be regarded as a kind of epistaxis. Then, too, it must be borne in mind that congestion and hæmorrhage may be set up by ingesta and purgatives; or may result from passive portal congestion, due to congenital heart disease or respiratory obstruction. Care must be taken to exclude hæmatemesis due to blood swallowed, during parturition, or subsequently from a cracked nipple or sore mouth. Apart from these causes some of the cases are undoubtedly septic in origin and associated with fever and, perhaps, jaundice.

As a rule the bleeding starts in 12 to 36 hours after birth. It may begin at the sixth hour or be delayed until the sixth day, or even as late as the sixth week. Comparatively few cases begin after the first week. There are no premonitory symptoms. The bleeding is the first sign. The blood may be dark in colour at first, if it comes from the stomach or high up in the intestine, and may contain clots. If hæmatemesis is profuse or the bleeding takes place from the colon the blood is bright red, thin and watery. It may begin before food has been taken, or follow vomiting or the passage of mucus. The hæmorrhage may take place from the umbilicus only, or, indeed, from any single place; or there may be bleeding from several different places and into various organs, more or less simultaneously. This is especially apt to occur in septic cases. In mild cases the bleeding is slight, ceasing in a few hours or in a day or two. Others are more serious and prolonged. As a rule, the babe dies or recovers in three or four days. Rarely, there is a single attack of hæmatemesis. The greater and the more sudden the hæmorrhage, the worse is the prognosis. It is bad also if the blood is coming from a mucous membrane and is bright red, indicating continuous oozing; if there is high fever and jaundice indicative of sepsis; if there is a large spleen or a history of syphilis; or if there is diarrhœa. The mildest cases are those of bleeding from the umbilicus, for local remedies can be easily applied. The mortality varies from 35 to 78 per cent. The child becomes profoundly anæmic, collapsed, and dies from asthenia or in convulsions.

For local bleeding recourse must be had to adrenalin and calcium chloride, or to sterile gauze soaked in ten per cent. solution of gelatin. For gastro-intestinal hæmorrhage give gelatin and whey, or gelatin and water, by the mouth; or a 2-5 per cent. solution by the rectum, with a long tube; or, in very dangerous cases, inject a 2 per cent. solution *sub cutem* in doses of 10-15 c.c., three to four times daily, or every three hours up to three or four doses. The fluid must be boiled for six hours. It may cause toxic symptoms, in consequence of toxins present, and has been known to give rise to tetanus. Hazeline in doses of 10 minims, or calcium chloride or lactate in doses of 2 grains, should be given by mouth, three or four hourly. Bismuth is worth a trial, and a hot bath may be of assistance by dilating the superficial vessels. Keep the child warm, and avoid irritating food. On the whole, it is doubtful whether treatment is of very great service except in local bleeding and in mild cases, which might get well independently.

